# The genome-wide transcriptional regulatory landscape of ecdysone in the silkworm

**DOI:** 10.1186/s13072-018-0216-y

**Published:** 2018-08-27

**Authors:** Dong Cheng, Tingcai Cheng, Xi Yang, Quan Zhang, Jianfeng Fu, Tieshan Feng, Jiao Gong, Qingyou Xia

**Affiliations:** 1 State Key Laboratory of Silkworm Genome Biology, Southwest University, Chongqing, 400715 China; 2 Chongqing Engineering and Technology Research Center for Novel Silk Materials, Southwest University, 2, Tiansheng Road, Beibei, Chongqing, 400715 China

**Keywords:** Histone modification, Enhancer, Ecdysone receptor, Regulatory element, Silkworm

## Abstract

**Background:**

The silkworm, *Bombyx mori*, a typical representative of metamorphic insects, is of great agricultural and economic importance. The steroid hormone ecdysone (20-hydroxyecdysone, 20E) is the central regulator of insect developmental transitions, and its nuclear receptors are crucial for numerous biological processes, including reproduction, metabolism, and immunity. However, genome-wide DNA regulatory elements and the ecdysone receptor (EcR) that control these programs of gene expression are not well defined.

**Results:**

In this study, we investigated the alterations in three types of histone modification in silkworm embryonic cells treated with 20E by chromatin immunoprecipitation sequencing (ChIP-seq). We identified enhancers using histone modifications and derived genome-wide ecdysone-dependent enhancer activity maps in the silkworm. We found enhancers enriched for monomethylation of histone H3 Lys4 (H3K4me1) that showed dynamic changes in acetylation of histone H3 Lys27 (H3K27ac) after 20E treatment and functioned to regulate the transcription of specific genes. EcR regulated transcription by binding not only to proximal promoters but also to the distal enhancers of target genes. Moreover, only 52.65% EcR peaks contained ecdysone response element (EcRE) motif, suggesting that EcR regulates the expression of target genes not only by binding directly to EcRE, but also by binding with other transcription factor.

**Conclusions:**

Our findings provide novel insights into the complex regulatory landscape of hormone-responsive cell activity and a basis for understanding the complex transcriptional regulatory processes of ecdysone.

**Electronic supplementary material:**

The online version of this article (10.1186/s13072-018-0216-y) contains supplementary material, which is available to authorized users.

## Background

Spatial–temporal specificity in gene expression is achieved according to information in cis-acting DNA regulatory elements including insulators, promoters, and enhancers [1, 2]. Enhancers have emerged as key cis-regulatory elements, independent of their orientation or distance, to affect gene transcription [3–5]. An emerging view is that enhancers can recruit transcription factors (TFs) to specific binding sites, and specific combinations of TFs and their co-activators and co-repressors result in gene activation or repression [6, 7]. In addition, enhancers are becoming increasingly appreciated as the sites of functional variation within the genome that contribute to diverse diseases [4]. However, the vast majority of enhancers in animal genomes and their spatiotemporal activities are unknown as suggested by the small number of gene expression patterns that have been linked to specific enhancers [8, 9]. Thus, there is substantial interest in identifying enhancers and in elucidating how they control activity-dependent gene transcription.

Post-translational modifications of histones, such as acetylation and methylation of lysine residues of histone H3, play important roles in regulating gene expression by altering the chromatin structure [10]. Genome-wide mapping of histone modifications has revealed highly stereotypical patterns, with different marks enriched at regulatory elements [8, 9, 11–13]. For example, in mammals and insects, trimethylation of histone H3 Lys4 (H3K4me3) and acetylation of histone H3 Lys27 (H3K27ac) have been associated with active promoters [10, 14], and monomethylation of histone H3 Lys4 (H3K4me1) and H3K27ac have been detected in active enhancers [10, 15, 16]. Therefore, histone-modified marks allow the genome-wide prediction of regulatory elements.

Although enhancer elements are known to be associated with certain histone modifications, the relationship of these modifications with gene expression has not been clearly defined [17–19]. H3K4me1 modification-based prediction of enhancers has been widely used, but a significant percentage of the enhancers were inactive when tested in reporter assays, suggesting that many of them might not function to promote activity-regulated transcription [10, 20]. Moreover, several lines of evidence indicate that H3K4me1 can mark active enhancers as well as those in a poised state [15, 21]. Poised enhancers contain information about the future developmental potential of cells, as well as their ability to respond to external stimuli [22–24]. Several studies have shown that the subset of H3K4me1-enriched enhancers that also have H3K27ac enrichment are actively engaged in regulating transcription. In addition, H3K27ac has been shown to be a reliable marker for active enhancers in mammalian systems and in *Drosophila* [15, 22]. These studies suggested that the presence of the H3K27ac modification might mark enhancers and that putative enhancers display an increase in H3K27ac in response to stimuli that are functionally engaged in regulating gene transcription. This may be useful in identifying functionally relevant activity-dependent enhancers.

The silkworm, *Bombyx mori*, a typical representative of metamorphic insects, is of great agricultural and economic importance [25]. The steroid hormone ecdysone (20-hydroxyecdysone, 20E) plays an important role during major developmental transitions and is crucial for various important biological processes in metamorphic insects [26–28]. The molecular mechanisms underlying the transduction of a hormonal signal into a transcriptional response have been well studied [29–33]. The active form of ecdysone binds to a heterodimer of the ecdysone receptor (EcR) and ultraspiracle (USP) to form a functional receptor and triggers the transcription of primary and secondary response genes in specific tissues [26, 34]. The EcR/USP complex binds to ecdysone response elements (EcREs), specific sequences near ecdysone-responsive target genes, to regulate gene transcription [35]. However, functional studies of cis-regulatory elements have been restricted to a small number of sequences that were originally identified by promoter analysis of ecdysone-induced genes. For example, *Hsp27*, *Eip28/29*, *Fbp1*, and *Sgs*-*4* are induced directly by ecdysone and contain an EcRE in their basal promoter region [36–38]. The total number of identified ecdysone-responsive enhancers is not comparable to the hundreds of genes that are regulated by the hormone in different cell types. Because of the lack of a comprehensive map of cis-regulatory elements, it has remained unclear how a single hormone via its nuclear receptor can elicit different regulatory and physiological responses in different cell types [39]. While the interactions between ecdysone and its responsive transcription factors have been well characterized at the molecular level [30], the mechanism of EcR regulation of ecdysone-responsive gene expression at the genome level remains unclear.

In this study, we carried out chromatin immunoprecipitation sequencing (ChIP-seq) with antibodies for EcR and three different histone marks to identify ecdysone-responsive regulatory elements, using silkworm embryonic (BmE) cells treated with 20E. We then correlated the histone profiles with genome-wide gene expression levels obtained by RNA sequencing (RNA-seq), to infer functional states of histone modifications and to link the regulatory elements to their target genes. We expected this integrative analysis to provide novel insights into the complex regulatory landscape of hormone-responsive cell activity.

## Results

### Identification and characterization of H3K4me1, H3K4me3, and H3K27ac sites

To investigate genome-wide alterations in histone modifications and to identify ecdysone-responsive enhancers in silkworm BmE cells, we performed ChIP-Seq using antibodies against H3K4me1, H3K4me3, and H3K27ac before and after 20E treatment. Previous studies have shown that H3K4me3 is an active mark that is often found in the proximal promoter region, while H3K4me1 is generally associated with enhancers located in the distal promoter region [18, 19]. H3K27ac has been shown to selectively mark active regions. In total, we identified 10,768 H3K4me3 peaks, 7266 H3K4me1 peaks, and 17,487 H3K27ac peaks after 20E treatment, respectively (Additional file 1).

**Fig. 1:**
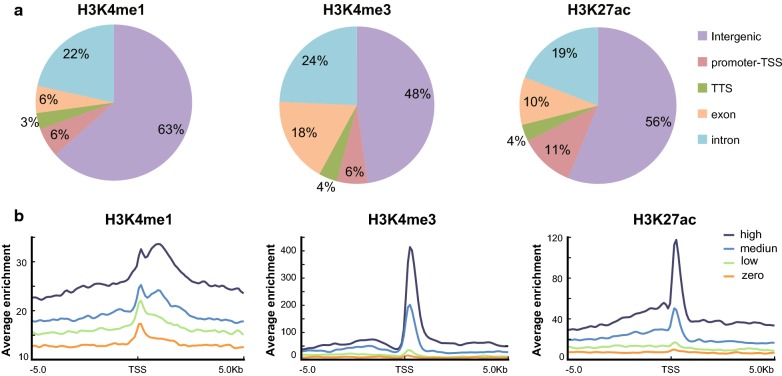
Genome-wide distribution of H3K4me1, H3K4me3, and H3K27ac modifications. **a** Pie charts showing the distributions of H3K4me1, H3K4me3, and H3K27ac across the genome without 20E treatment. “Promoter-TSS” indicates the region − 1 kb to + 200 bp of the TSS. **b** Average ChIP-seq signal profiles for genes with different expression levels were generated for the histone modifications around the TSS without 20E treatment. Genes were divided into four categories according to their mRNA levels: no expression, low-level expression, medium-level expression, and high-level expression

We determined the distribution of each histone modification at the genome level (Fig. 1a). Of the peaks identified for H3K4me3, 5599 (52%) intersected with annotated genes or their proximal promoters (here defined as regions located 1.5 kb upstream and downstream of transcription start site) and nearly half of peaks (48%) corresponded to the intergenic regions. In the case of H3K4me1, only 37% (2688) of the peaks intersected with genes or their proximal promoters, and most peaks appeared in the intergenic regions, which is consistent with the fact that H3K4me1 is generally associated with distal cis-regulatory elements [40]. H3K27ac was mainly distributed in the intergenic and proximal promoter regions, between H3K4me1 and H3K4me3. The genomic distribution of all of three histone marks did not show significant changes after 20E treatment (Additional file 2a), which suggests that the genomic distribution of these histone marks is quite stable.

Composite profiles of each histone modification around the transcription start site (TSS) were generated according to the expression level of the corresponding gene (Fig. 1b). H3K27ac and H3K4me3 signals were enriched around the TSS and were positively correlated with the gene expression levels. In contrast, H3K4me1 signal was lower in the 2 kb upstream or downstream of the TSS, and the peak signals were also positively correlated with the gene expression levels. No differences in the distribution patterns of these histone modifications were observed after 20E treatment (Additional file 2b). The distribution profiles of H3K27ac, H3K4me3, and H3K4me1 in our ChIP-seq analysis were consistent with the well-known distribution pattern of histone modifications, indicating strong reliability of our ChIP-seq data [41, 42].

### Genome-wide changes in histone modifications induced by treatment with 20E

**Fig. 2:**
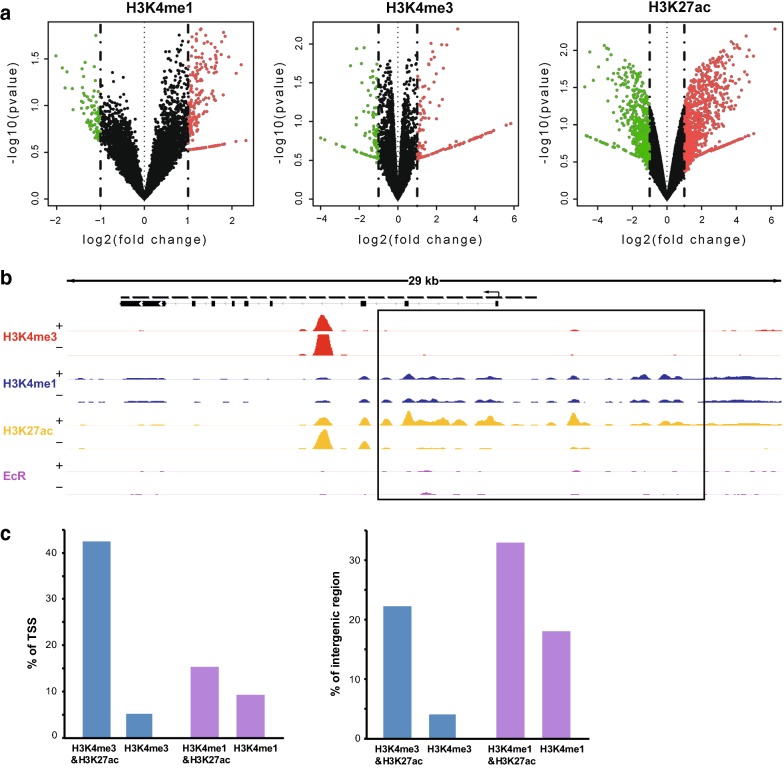
Genome-wide alterations in histone modification statuses after 20E treatment. **a** Histone modification statuses are altered throughout the genome after 20E treatment. Regions showing a more than twofold increase (red dots) or decrease (green dots) in signals between 20E- and DMSO-treated groups were defined as having increased or decreased histone modifications, respectively. Dark dots represent the regions that showed less than twofold changes. The p value of ordinate from DEseq 2 represented the probability of difference. **b** IGV genome browser tracks of the *Hr3* locus with data for indicated histone modifications and RNA-seq. “+” and “−” indicate the presence and absence of 20E treatment, respectively. The square indicated the region that H3K27ac modification changed drastically after 20E treatment. **c** Histone modification collaboration in different genome regions. “H3K4me3&H3K27ac” represented the H3K4me3 sites that overlapped with H3K27ac sites. “H3K4me1&H3K27ac” represented the H3K4me1 sites that overlapped with H3K27ac sites. “H3K4me3” represented the H3K4me3 sites that did not overlap with H3K27ac sites. “H3K4me1” represented the H3K4me1 sites that did not overlap with H3K27ac sites

To examine whether histone modification statuses were altered throughout the genome by treatment with 20E, we firstly report the overlap of called peaks between 20E-treated BmE cells (“Treated”) and DMSO-treated BmE cells (“Control”). The results showed that about 60% of H3K4me1 sites overlapped before and after 20E treatment, and corresponding to 95.4% of H3K4me3 sites and 84% of H3K27ac sites. It’s worth noting that the number of altered H3K4me1 sites was similar to altered H3K27ac sites. Besides, histone modification signals were searched between 20E-treated and DMSO-treated BmE cells, and the differences in signals based on direct overlap of called peak were evaluated (Fig. 2a). A number of regions with altered signals of H3K27ac by 20E treatment were observed. On the other hand, there were few regions in which the signals of H3K4me3 and H3K4me1 were altered by 20E treatment. For example, compared to H3K4me3 and H3K4me1, H3K27ac was increased at the nuclear receptor *HR3* locus (Fig. 2b). The *HR3* is essential for developmental switches during insect development and metamorphosis regulated by ecdysone [43, 44]. A substantial increase in H3K27ac at the *HR3* regulatory regions was correlated with an increase in *HR3* mRNA (Gene ID: BMgn009688). These results suggested that H3K27ac changed more dynamically after 20E treatment, and the activity-dependent increase in H3K27ac at the *HR3* locus might be required for *HR3* transcription.

The distributions of H3K4me1, H3K4me3, and H3K27ac suggested that these three modifications might differentially collaborate in different genome regions (Fig. 1a). We firstly calculated the overlap between each histone modification after 20E treatment (Additional file 3). Nearly 28.9% and 52.3% H3K27ac sites overlapped with H3K4me1 and H3K4me3 sites, respectively. A relatively small amount of H3K4me1 sites colocalizated with H3K4me3 sites. We also determined the degree of co-occurrence of H3K4me1 and H3K4me3 at the TSSs of all genes and of H3K4me1 and H3K27ac for all intergenic regions throughout the genome (Fig. 2c). Among all TSSs, approximately 6746 (42.90%) were marked by both H3K27ac and H3K4me3, while only about 2359 (15%) TSSs were marked by both H3K27ac and H3K4me1. This suggested that H3K4me3 modification preferentially might collaborate with H3K27ac modification at or near TSSs. In the intergenic regions, about 4011 regions (33.2%) were marked with both H3K27ac and H3K4me1 and 963 regions (17.25%) were marked only with H3K4me1. In contrast, approximately 1699 regions (22%) were marked by both H3K27ac and H3K4me3, which demonstrated that H3K4me1 modification preferentially collaborates with H3K27ac modification in intergenic regions. Together, these results showed differential collaboration of H4K4me3, H3K4me1, and H3K27ac in different genomic regions and suggested that synergy of histone modifications might be useful to identify transcriptional regulatory elements.

### Identification and characterization of enhancers

**Fig. 3:**
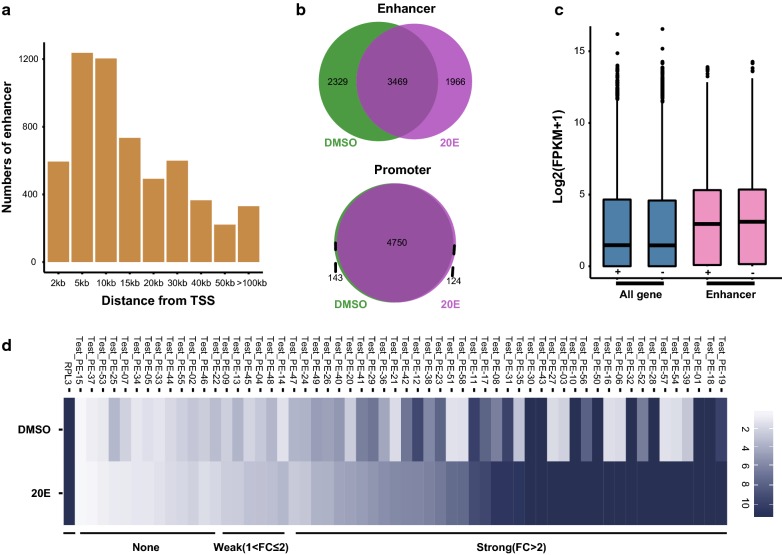
Genome-wide enhancer analysis and functional verification. **a** Distance between the enhancer and the TSS of the nearest gene. **b** Overlap of enhancers and promoters before and after 20E treatment. **c** Average expression level changes of enhancer target genes and all genes. “+” and “−” indicate the presence and absence of 20E treatment, respectively. **d** Validation of putative enhancers by luciferase assays. We named these 58 candidate enhancers with “Test_PE” as a prefix, respectively. All results are displayed as a fold increase over the PGL3 reporter backbone with hsp70 core promoter. Hsp70 core promoter and Rpl3 enhancer combination, labeled as RPL3, were used as positive controls. Strongly induced enhancers were divided into three classes: (1) 20E-independent, where the addition of 20E did not change enhancer activity; (2) 20E-increased, where 20E increased enhancer activity; (3) 20E-decreased, where 20E decreased enhancer activity

Enhancer prediction on the basis of histone modification marks is currently widely used, and recent genome-wide studies have shown that enhancers are DNA sequences that are marked with H3K4me1 modification and promoter regions can be defined as regions with H3K4me3 modification [20, 45]. Based on these studies, we defined enhancers as distal regulatory elements (DRE) harboring H3K4me1 binding sites that were 1.5 kb away from TSSs. Promoters were defined as DNA regions with H3K4me3 binding sites that were located within 1.5 kb from TSSs. We identified 5841 DREs and 4931 putative promoter elements after 20E treatment (Additional file 4). The DREs we defined were mainly distributed in the intergenic regions (Additional file 5a), similar to the distribution of the H3K4me1 modification. The distances between DREs and the TSSs of the nearest genes varied greatly, mainly from 5 to 10 kb (Fig. 3a), which is consistent with the fact that DREs can function at variable distances upstream or downstream of target genes. Notably, some DREs were located more than 100 kb from the TSS of their target gene, and they presumably form a promoter–enhancer loop by chromatin folding that regulates gene transcription [46, 47]. We found that 41.1% of DREs were specifically modified versus only 4.1% of promoters after 20E treatment (Fig. 3b). This indicated that DREs changed dynamically after 20E treatment, and genes might be regulated mainly by DREs in cells stimulated with ecdysone.

We assigned DREs to their nearest genes and quantified the expression of these genes using RNA-seq data. The results showed that the average expression of the genes nearest to the DREs was higher than the average expression of all genes (Fig. 3c). This indicated that the presence of the DREs identified in this study correlated well with increased activity of the nearest genes. We randomly selected 58 candidate enhancers from DREs for validation by luciferase assays before and after 20E treatment. The data was supplemented in Additional file 6. As enhancers were defined as H3K4me1 sites that were devoid of H3K4me3 modification, all of 58 enhancer elements were H3K4me1-positive and H3K4me3-negative. The results showed that 46 out of the 58 (79.3%) candidate enhancers could increase reporter expression, and 39 out of the 58 (57.7%) candidate enhancers increased significantly high reporter expression (≥ twofold) (Fig. 3d). We observed that the activities of these 39 strong enhancers were differentially affected by ecdysone. For example, the enhancers of *Vrille* and *Laminin* showed reduced activity after 20E treatment. *Vrille* was a circadian rhythm-related gene and *Laminin* encoded a structural protein. Both of them were critical to cell structure and physiological activities. 20 enhancers among 39 strong enhancers increased reporter gene expression levels upon 20E treatment, including those of the ecdysone response genes *EcR* and *Eip75B*. It was worth noting that most these 20 enhancers were H3K27ac-positive only after 20E. Besides, 17 enhancers were active at similar levels before and after 20E treatment, including the enhancers of Se*rpin14* and *zfh1*. Together, these results indicated that enhancers can enhance gene expression to varying degrees, and their activities are modulated by the steroid hormone ecdysone.

### Ecdysone regulates the dynamics of H3K27ac at enhancers

**Fig. 4:**
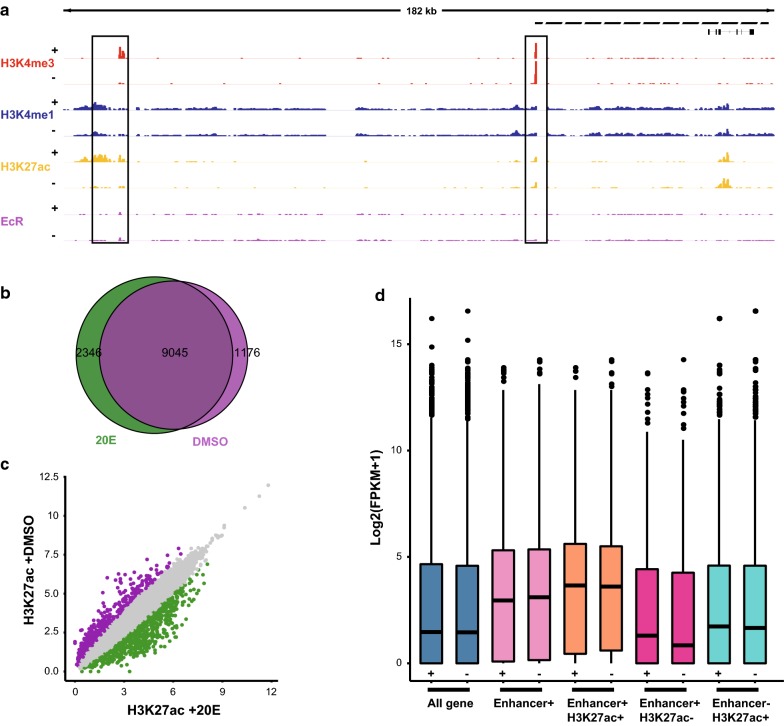
Dynamics of H3K27ac at enhancer elements. **a** Representative locus (*Br*-*C* locus) demonstrating distinct H3K27ac dynamics at enhancers in response to ecdysone hormone activity. “+” and “−” indicate the presence and absence of 20E treatment, respectively. **b** Classification of putative enhancers by distinct H3K27ac dynamics in response to ecdysone activity. Green indicates an at least twofold increase in H3K27ac signal and violet indicates an at least twofold decrease in H3K27ac signal. H3K27ac with a less than twofold change is represented in grey. **c** Changes in average enrichment of H3K27ac before and after 20E treatment. **d** Average expression of the nearest gene to enhancers with and without H3K27ac enrichment. “+” and “−” indicate the presence and absence of 20E treatment, respectively

H3K27ac modification and DREs were dramatically altered after 20E treatment (Figs. 2a, 3b). Thus, we investigated the pattern of H3K27ac at known ecdysone-responsive enhancers. *Broad*-*Complex* (*Br*-*C*) is an early ecdysone-responsive gene encoding a family of zinc-finger transcription factors, which play an important role in the metamorphosis of insects (Fig. 4a). Activity-dependent induction of *Br*-*C* transcription is mediated by two experimentally confirmed proximal and distal regulatory elements [48]. We found a substantial increase in H3K27ac enrichment in the distal regulatory element, which correlated with an increase in *Br*-*C* mRNA. This indicated that the steroid hormone ecdysone regulates the enrichment of H3K27ac at DREs, which might be a key step in gene expression.

We filtered out H3K27ac peaks that were located within regions of 1.5 kb from TSSs, which likely represented promoters, and we analyzed the genomic distribution of the remaining H3K27ac peaks before and after 20E treatment (Fig. 4b). The results showed that 2346 distal H3K27ac peaks were detected only after 20E treatment, which indicated that ecdysone regulates the dynamics of distal H3K27ac. Genome-wide quantification of H3K27ac levels at DREs revealed that 741 DREs exhibited an at least twofold increase in H3K27ac after 20E treatment (Fig. 4c). Most DREs exhibited a stable level of H3K27ac before and after 20E treatment. We also identified 572 sites that underwent a decrease in H3K27ac after 20E treatment. For example, a significant decrease in H3K27ac enrichment upstream of the nuclear receptor *FTZ*-*F1* after 20E treatment was observed (Additional file 5b). The *FTZ*-*F1* gene is expressed in most cells in a temporally specific manner and plays important roles during embryogenesis, larval ecdysis, and early metamorphic stages [49, 50]. The decrease in H3K27ac was consistent with previous reports that *FTZ*-*F1* expression is induced immediately after the decline in the ecdysone level. Together, these results showed that H3K27ac changed dynamically at DREs after 20E treatment, which indicated that the enrichment of H3K27ac at DREs might determine which among the thousands of DREs are functionally engaged in driving ecdysone-responsive gene transcription.

To determine whether H3K27ac enrichment at DREs are indeed capable of stimulating activity-dependent transcription, we compared gene expression among various patterns of H3K27ac modification, including DREs with H3K27ac modification, DREs without H3K27ac, and H3K27ac sites that did not overlap with DREs. We assigned regulatory elements from each of these groups to the nearest gene and quantified the expression of these genes using RNA-seq data. We found that the expression levels of the genes nearest to the DREs strongly correlated with the levels of H3K27ac in their respective DREs both before and after 20E treatment (Fig. 4d). The average expression of the genes the closest to DREs with H3K27ac enrichment was higher than that of genes the closest to DREs without H3K27ac enrichment. When taking all DREs in consideration, the average expression of their nearest genes was also higher than that of genes the closest to DREs without H3K27ac enrichment. This indicated that H3K27ac enrichment is a deterministic feature of active DREs and that H3K27ac enrichment at DREs activates transcription of ecdysone-responsive genes. H3K27ac sites that did not overlap with DREs also exhibited a correlation with activity-dependent expression of the nearest gene, suggesting that these sites might also act as putative enhancers under the given chromatin environment.

We investigated the functions of the nearest genes associated with each of the above patterns of H3K27ac modification using Gene Ontology (GO) analysis (Additional file 5c). We found that the nearest genes for all DREs were enriched for processes related to signal transduction and energy metabolism. The genes closest to the DREs with H3K27ac enrichment were enriched for lipid metabolism and DNA biosynthesis, which are associated with ecdysone activity [51]. Some ecdysone-responsive genes, such as *Eip74*, *Eip75*, and *Br*-*C*, were enriched in these pathways. The genes closest to the DREs without H3K27ac enrichment were less enriched for genes related to the ecdysone signal pathway. This result indicated that H3K27ac enrichment at DRE stimulates ecdysone-dependent gene transcription. Taken together, DREs are functionally engaged in regulating gene transcription by the enrichment in H3K27ac in response to ecdysone activity.

### EcR binds regulatory elements to regulate ecdysone-responsive genes

**Fig. 5:**
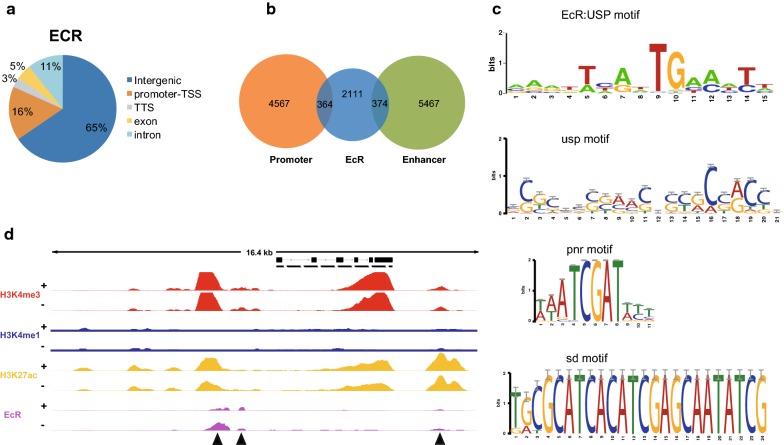
EcR regulates gene transcription through binding to both proximal and distal regulatory elements. **a** Pie charts showing the distribution of EcR-enriched regions across the genome after 20E treatment. “Promoter-TSS” represents the egion -kb to 200 bp of the TSS. **b** EcR overlaps with promoter and enhancer elements, respectively. **c** Motif analysis of EcR-enriched regions by a MEME-ChIP. **d** IGV genome browser screenshots of EcR, histone modification, and strand-specific RNA-seq tracks for the arginine methyltransferase gene locus

To determine how *cis*-regulatory elements modulate ecdysone-responsive gene expression, we performed ChIP-seq with an antibody against EcR using BmE cells after 24 h of 20E treatment. We identified 2849 peaks after mapping the EcR-binding regions to the silkworm genome (Additional file 7). Relative to the 16% of EcR peaks that were mapped to proximal promoter (− 1 kb to + 200 bp) regions, a large fraction of the EcR peaks (65%) were distributed in the intergenic regions (Fig. 5a). We then mapped the locations of EcR peaks to promoter and DREs regions: 374 and 376 EcR peaks overlapped with DREs and promoter elements, respectively (Fig. 5b). Some EcR peaks were located in the DREs of known ecdysone-responsive genes, such as *Eip75*, *Br*-*C*, and *CYP450*. These results suggested that EcR can regulate target gene expression not only by binding to proximal promoters, but also by binding to DREs.

Previous studies have shown that EcR binds to the EcRE sites of genes to activate their transcription. We found that 1417 EcR peaks (52.65%) were located in a region that contained at least one EcRE motif (Fig. 5c and Additional file 8). In these regions, we also identified the USP-binding motif (Fig. 5c); USP binds with EcR to form a heterodimeric receptor complex. This indicated that functional EcR/USP heterodimer directly binds to EcREs of ecdysone-responsive genes to induce transcriptional activation. However, approximately half of the regions associated with EcR peaks did not contain an EcRE motif. In contrast, we found that the motif of TALE-type homeodomain transcription factors, pnr, and that of TEA domain transcription factors, sd, were significantly enriched in these EcR peak-associated regions (Fig. 5c). For example, we identified three EcR peaks in the arginine methyltransferase locus (Fig. 5d), of which only the most downstream EcR peak was enriched for the EcRE motif. Motifs of homeodomain factors, such as mirr and ptx1, were enriched in two other EcR peak regions (Additional file 9a). This indicated that parts of EcRs might bind with other factors to form a complex instead of binding to EcRE to directly regulate gene expression. In addition, we observed that the average expression of genes associated with the two types of EcR peaks was similar and higher than the average expression of all genes (Additional file 9b). Taken together, these data suggested that EcR regulates the expression of target genes not only by directly binding to EcREs in the regulatory regions, but also by cooperating with other transcription factors.

## Discussion

In this study, we used ChIP-seq to characterize the alterations in three histone modifications induced by the presence of ecdysone in silkworm embryonic cells and to generate an ecdysone-responsive regulatory element map for the silkworm. We demonstrated that most of the DREs identified could induce reporter expression and were consistent with target gene expression. The enrichment of H3K27ac at DREs in response to 20E treatment might facilitate gene expression. Our results indicated that EcR could bind not only proximal promoters, but also DREs. Moreover, nearly half of the genome regions associated with EcR peaks contained EcRE motifs, and those without EcRE motifs might bind with other transcription factors instead of directly binding to EcR to regulate gene expression, which reflected the complexity of ecdysone-mediated gene transcription.

The identification of stimulus-responsive enhancers by genomic mapping of certain chromatin features has provided important insights into spatiotemporal patterns of gene expression [52]. Given the widespread use of histone modifications to predict enhancers, it is interesting that there is no consensus about which marks should be used [45]. Although our functional assay demonstrated that most of the regions displaying H3K4me1 marks are associated with enhancers, one-third of the H3K4me1-marked regions assayed exhibited no signs of enhancer activity (Fig. 3d). These sequences could be promoter-specific enhancers or enhancers that act in concert with other sequences not tested in our assays. These results imply that none of the known modifications correlates perfectly with enhancer activity, and even combinations of marks are not perfect predictors [18]. Thus, it will be interesting to learn about the functional roles of such marks and their combinations, and how these will improve enhancer predictions. In addition, 11 out of the 58 enhancers identified by our luciferase assay exhibited activity only upon 20E treatment (Fig. 3d). This result suggested that enhancer activity depends on specific conditions and certain putative enhancers become active only following a specific stimulus. Furthermore, we scanned all of these 58 enhancer elements sequence for EcR motif enrichment and found that 41.3% of ecdysone-responsive enhancers contained EcR motif relative to 25% of enhancers that did not respond to ecdysone. This result indicated that the ecdysone-responsive enhancers were more likely to exhibit EcR binding or an EcR motif. However, further evidence needs to be provided in future studies.

Our systematic genome-wide study of histone modifications provided several surprising insights into transcriptional regulation. Although H3K27ac modifications have been shown to be present at a subset of enhancers, the relationship between them remained unclear [4]. Our data showed that the enrichment of H3K27ac at enhancers changed dynamically, and the enrichment of H3K27ac at enhancers was consistent with high expression of the nearest genes (Fig. 4d). Our results supported the existence of poised enhancers that have been previously reported, and elements existing in a poised state were activated after 20E treatment [15]. We could discriminate between active and inactive enhancer regions on the basis of H3K27ac enrichment, thus obtaining more general information about the activity of cells in response to ecdysone hormone. These data are compatible with a temporal scenario in which poised enhancers marked by H3K4me1 acquire H3K27ac when activated and lose this mark when inactivated [14, 23, 24]. However, the confirmation of this scenario requires further study.

Ecdysone signals are important regulators of insect development during molts and metamorphosis. Ecdysone activates a number of ecdysone-responsive genes through a heterodimeric receptor complex [34]. Interestingly, some of the most strongly up-regulated genes, including the known ecdysone targets *Eip75*, *br*, *Eip74*, *Hr46*, and *E23*, appeared to be induced via multiple ecdysone-responsive enhancers. This showed that enhancer strength correlates well with gene expression and suggested that strong induction was mediated via multiple enhancers [53]. Interestingly, some of the strongly up-regulated genes, such as *Eip93*, appeared to be strongly regulated via a single enhancer. Previous studies have shown that EcREs are mainly located in the promoter regions and bind directly with EcR/USP complex to regulate the transcription of ecdysone-responsive genes [54, 55]. Our results showed that the EcR-binding regions were identified not only in promoter regions of target genes, but also in DREs (Fig. 5b). Relative to proximal EcR-binding regions, motifs in distal elements varied greatly, and different classes of factors were enriched, including the C2H2 zinc finger factor, CTCF, the homeodomain factor, Optix, the Fork head/winged-helix factor, br, the SMAD/NF-1 DNA-binding domain factor, Mad, and the SAND domain factor, Deaf1 (Additional file 9c). These transcription factors are encoded by different gene families and might be consistent with the different functions of distant enhancers [56, 57]. Moreover, numerous EcR peaks were identified in intergenic regions and they did not overlap with promoters and DREs (Fig. 5a, b). For example, we found several EcR peaks 3 kb upstream of the *fisd* gene, and no DREs were identified within this region. This supports that EcR can bind to other types of regulatory elements, such as silencers to regulate target gene expression [58]. Half of the EcR peaks were in regions that contained EcRE motifs, indicating target gene expression regulation by direct binding. Nearly half of the EcR peaks contained binding motifs for other transcription factors, which might firstly bind with EcR to form a complex and then bind to regulatory regions of target genes. Similarly, among 39 strongly induced enhancers that were validated by the reporter assay (Fig. 3d), 17 enhancers contained EcRE motifs and 22 enhancers without EcRE motifs contained motifs for other transcription factors. This indicated that EcR can regulate target gene expression in a direct and an indirect manner. Combined, our results show that ChIP-seq in combination with 20E treatment has strong potential in identifying novel genome regions associated with ecdysone response.

## Methods

### Cell culture

Silkworm embryo-derived BmE cells were cultured in Grace’s insect medium (Gibco, Gaithersburg, MD, USA) supplemented with 10% fetal bovine serum at 27 °C. Unless stated otherwise, we used 20E (Sigma, St. Louis, Missouri, USA) at a concentration of 41 mM and an incubation time of 24 h.

### RNA-seq

Approximately 10^6^ BmE cells were treated with DMSO or 20E for 24 h in triplicate. Total RNA was isolated using TRIzol reagent (Invitrogen) according to the manufacturer’s protocol and was treated with DNase I. Purified RNA was prepared for sequencing with the Illumina mRNA-seq Sample Prep Kit. Samples were sequenced on an Illumina HiSeq 2500 (150-bp paired-end reads). For each sample, more than 25 million clean reads were obtained.

The quality of the raw and processed reads was evaluated using FastQC (version 0.11.6). Raw data were processed using Trimmomatic (version 0.36) to remove reads containing adapter sequences and low-quality reads [59]. We used STAR (version 2.5) to map the paired-end clean reads to the silkworm reference genome downloaded from KAIKObase (http://sgp.dna.affrc.go.jp/index.html) [60]. We retained only uniquely mapped reads with up to three mismatches. RSEM (version 1.3.0) was used to quantify the expression level of each gene [61]. We intersected the mapped reads with gene annotations from KAIKObase and calculated reads per kilobase million (RPKM) values of mapped reads (Additional file 10). Differential gene expression across the samples was analyzed using DESeq2 [62]. Differentially expressed genes were classified and analyzed by GO enrichment.

### ChIP-seq

Approximately 10^8^ BmE cells were treated with DMSO or 20E for 24 h. ChIP-seq was performed as previously described with minor modifications [63]. Briefly, the treated cells were fixed with 1% formaldehyde for 10 min at 37 °C. The fixed cells were lysed and the chromatin was sonicated to a size range of 200–600 bp. Solubilized chromatin was incubated with 10 μg antibody overnight at 4 °C. The following antibodies were used: anti-H3K27ac (17-683, Millipore), anti-H3K4me3 (17-614, Millipore), anti-H3K4me1 (07-436, Millipore), and anti-EcR (DDA2.7, Covance). Immunoprecipitated chromatin was washed, eluted, and subjected to cross-link reversal. Following RNase A and proteinase K treatments, the DNA was purified by phenol–chloroform extraction and ethanol precipitation. The ChIP DNA was prepared for sequencing using the TruSeq ChIP Library Preparation Kit (IP-202-1012; Illumina) according to the manufacturer’s protocol. Samples were PCR-enriched for paired-end sequencing on the Illumina HiSeq 2500 platform. For each sample, more than 20 million clean reads were obtained.

ChIP-seq data were pre-processed similar to RNA-seq data. We used bowtie2 (version 2.2.9) to map the paired-end clean reads to the silkworm reference genome downloaded from KAIKObase [64]. Only uniquely aligned reads were used. Highly enriched peaks were obtained by MACS2 using standard settings, allowing one modification (mfold = 20) [65]. For all ChIP-seq data sets, WIG files were generated with Bedtools (version 2.26.0), which were subsequently used for visualization purposes and for obtaining average signal profiles. Motifs were searched using MEME Suite 4.12.0 (http://meme-suite.org/) and RSAT peak-motifs with default parameters [66]. Compare discovered motifs with JASPAR core non-redundant insect (2018) databases (http://jaspar.genereg.net/).

### Identification of regions with altered histone modifications by 20E

The regions with altered signals of histone modifications induced by 20E were identified by comparing the ChIP-seq tag counts between the 20E-treated and DMSO-treated BmE cells. The differences in signals based on direct overlap of called peak were evaluated by DEseq2.

### Peak location

To determine the locations of H3K4me1, H3K4me3, H3K27ac, and EcR peaks, we firstly divided the whole genome into five unique regions according to gene set annotations (exon, intron, promoter-TSS (− 1 kb to + 200 bp TSS), TTS and intergenic region). Then, we assigned each peak to these regions. For some peaks that overlapped with several annotation regions, only peak summit sites were considered.

### Definition of enhancers based on ChIP-Seq data

We used H3K4me1 sites to define enhancers according to a previous study [67]. Excluding H3K4me1 sites that were located outside 1.5 kb of the TSS, the remaining H3K4me1 sites were defined as distal regulatory elements (DRE). DREs that overlapped with H3K27ac were defined as active DREs. Similarly, H3K4me3 sites that located within 1.5 kb of the TSS were defined as promoters, and promoters that overlapped with H3K27ac were regarded as active promoters. Target genes of enhancers were assigned according to their distance to the nearest gene TSS (using closestBed in Bedtools). The nearest genes were functionally analyzed using GO enrichment.

### Luciferase reporter assays

We randomly selected 58 H3K27ac-positive candidate enhancer elements after 20E treatment for luciferase assays. As enhancers were defined as H3K4me1 sites that were devoid of H3K4me3 modification, all of 58 enhancer elements were H3K4me1-positive and H3K4me3-negative. To test enhancer function in a reporter assay, we generated reporter constructs based on the pGL3-promotor backbone (Promega) by replacing the SV40 promoter sequence between *BglII* and *HindIII* with the core hsp70 promoter. Enhancers varied in size, and we used only approximately 1-kb regions around the center of enhancers as candidate enhancers. Candidate enhancers were PCR-amplified from silkworm genomic DNA, cloned into the reporter vector, and verified by Sanger sequencing. The primers used to amplify candidate enhancers are listed in addition files (Additional file 6). BmE cells (1 × 10^5^) were transfected with 110 ng of DNA, including 10 ng of Hrp3 vector as a transfection control, using X-tremeGENE HP DNA Transfection Reagent (Roche). After transfection for 24 h, the BmE cells were treated with 20E or DMSO for 24 h. Then, enhancer activity was measured by luciferase assay using the Dual luciferase kit (Promega) according to the manufacturer’s instructions. Four replicates were included for each candidate enhancer.

## Additional files

**Additional file 1.** ChIP-seq enriched peaks of H3K4me3, H3K4me1, and H3K27ac after 20E treatment.

**Additional file 2.** Genome-wide distribution of H3K4me1, H3K4me3, and H3K27ac modifications. **a** Pie charts showing the distribution of H3K4me-, H3K4me3-, and H3K27ac-enriched regions across the genome after 20E treatment. “Promoter-TSS” indicates the region − 1 kb to + 200 bp of the TSS. **b** Average ChIP-seq signal profiles for genes with different expression levels were generated for the histone modifications around the TSS after 20E treatment. Genes are divided into four categories according to their mRNA levels: no expression, low-level expression, medium-level expression, and high-level expression.

**Additional file 3.** The overlap of each histone modification sites after 20E treatment.

**Additional file 4.** Putative DREs (enhancers) and promoters were defined through modification-based prediction.

**Additional file 5.** **a** The distribution of DREs. **b** IGV genome browser tracks of the *FTZ*-*F1* locus with data for indicated histone modifications and RNA-seq. “+” and “−” indicate the presence and absence of 20E treatment, respectively. **c** GO-based gene functions for genes specifically associated with different types of enhancers. Red bars display functions based on nearest genes to H3K27ac-positive enhancers.

**Additional file 6.** The primers and histone modification status of 58 candidate enhancer elements were listed. The results of luciferase assays of candidate enhancers were also listed in the last few columns.

**Additional file 7.** ChIP-seq enriched peaks of EcR after 20E treatment.

**Additional file 8.** The list of EcR peaks containing EcRE motif.

**Additional file 9.** Motif analysis of EcR-enriched regions by MEME-ChIP and RSAT. **a** Motifs were enriched from EcR-binding sites of the arginine methyltransferase gene. **b** Average expression of the nearest genes of EcR. “EcR + EcRE” indicates EcR-peak regions enriched for the EcRE motif. “EcR − EcRE” indicated EcR-peak regions not enriched for EcRE motif. **c** Motifs enriched in distal EcR enhancer elements.

**Additional file 10.** RPKM values for RNA-seq in BmE cells after treated with 20E or DMSO.

## Abbreviations

DRE: distal regulatory elements
20E: 20-hydroxyecdysone
EcRE: ecdysone responsive element
EcR: ecdysone receptor
TFs: transcription factors
Br-C: broad-complex
USP: ultraspiracle
ChIP-seq: chromatin immunoprecipitation sequencing
RNA-seq: RNA sequencing
H3K4me1: monomethylation of histone H3 Lys4
H3K4me3: trimethylation of histone H3 Lys4
H3K27ac: acetylation of histone H3 Lys27
TTS: transcription termination site
TSS: transcription start site
FPKM: reads per kilobase million
RPKM: fragments per kilobase million
