# Supplementary figures and images for: The genome-wide transcriptional regulatory landscape of ecdysone in the silkworm
Source: Epigenetics Chromatin. 2018 Aug 27;11:48. doi: 10.1186/s13072-018-0216-y (PMC6109983; doi:10.1186/s13072-018-0216-y)

# Additional file 2

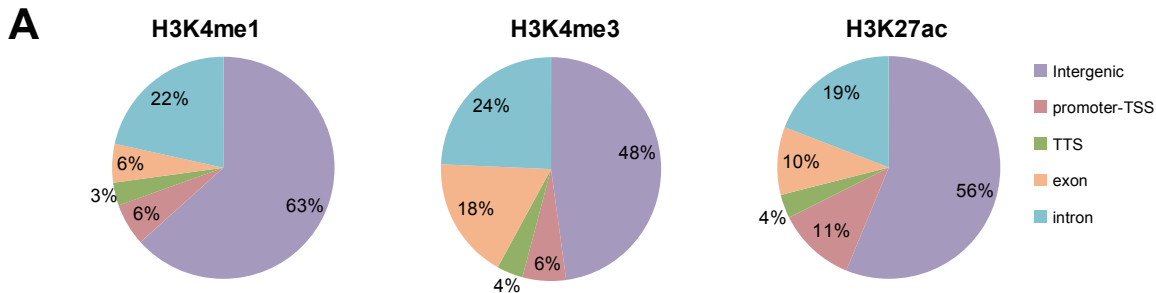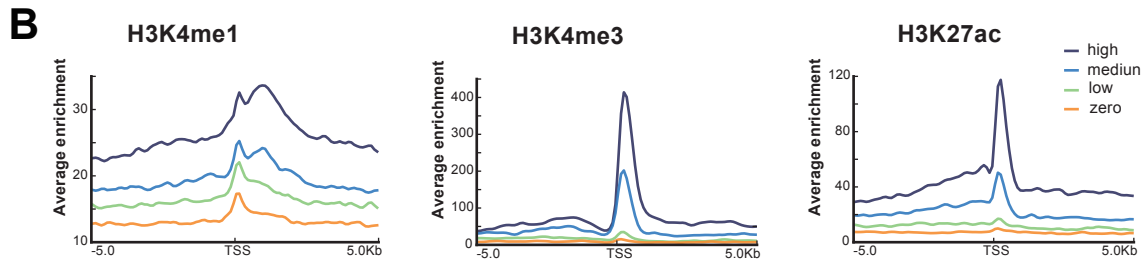

Supplement: Supplementary file 2 — Additional file 2. Genome-wide distribution of H3K4me1, H3K4me3, and H3K27ac modifications. a Pie charts showing the distribution of H3K4me-, H3K4me3-, and H3K27ac-enriched regions across the genome after 20E treatment. “Promoter-TSS” indicates the region − 1 kb to + 200 bp of the TSS. b Average ChIP-seq signal profiles for genes with different expression levels were generated for the histone modifications around the TSS after 20E treatment. Genes are divided into four categories according to their mRNA levels: no expression, low-level expression, medium-level expression, and high-level expression. [file 13072_2018_216_MOESM2_ESM.pdf]

# Additional file 3

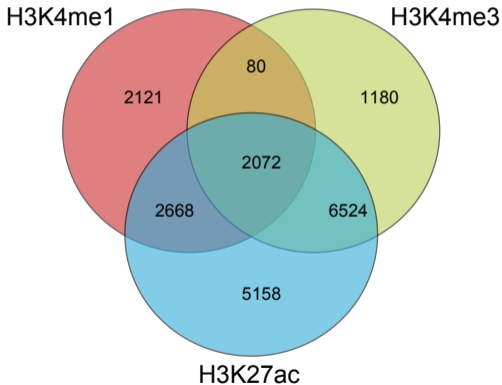

Supplement: Supplementary file 3 — Additional file 3. The overlap of each histone modification sites after 20E treatment. [file 13072_2018_216_MOESM3_ESM.pdf]

# 

**A**

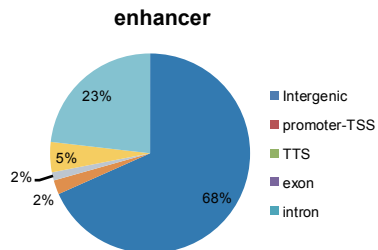

**C**

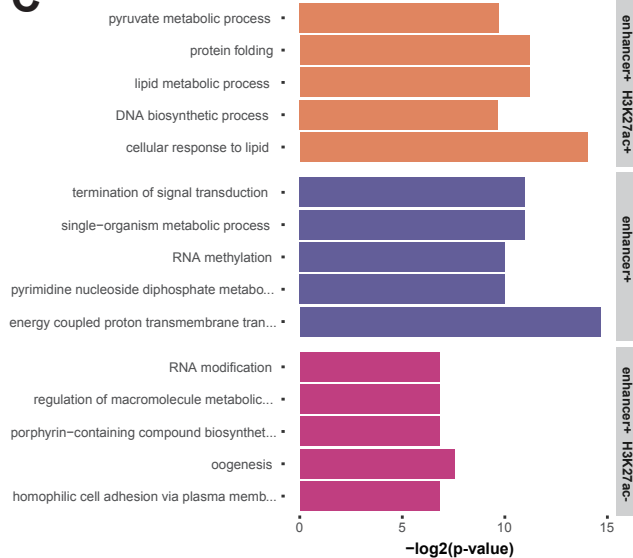

**B**

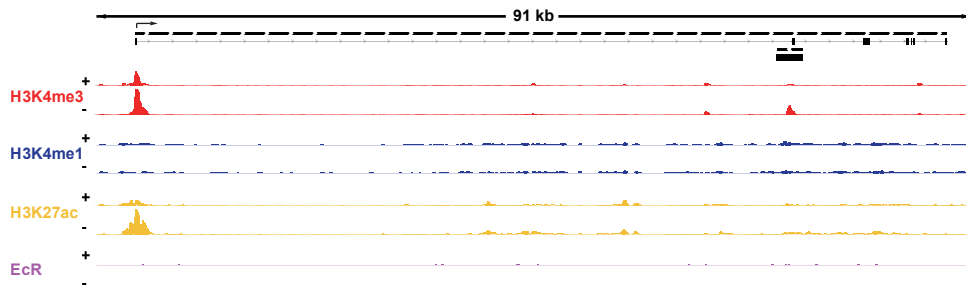

Supplement: Supplementary file 5 — Additional file 5. a The distribution of DREs. b IGV genome browser tracks of the FTZ-F1 locus with data for indicated histone modifications and RNA-seq. “+” and “−” indicate the presence and absence of 20E treatment, respectively. c GO-based gene functions for genes specifically associated with different types of enhancers. Red bars display functions based on nearest genes to H3K27ac-positive enhancers. [file 13072_2018_216_MOESM5_ESM.pdf]

# Additional file 9

**A**

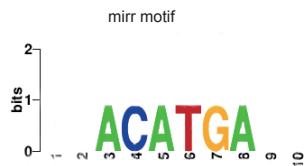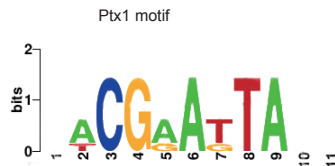

**B**

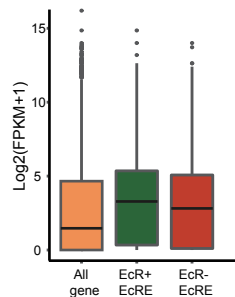

**C**

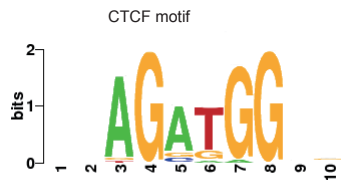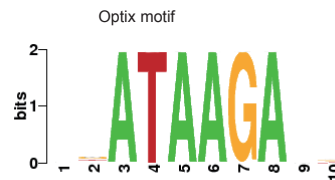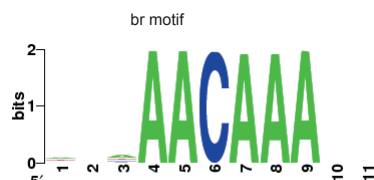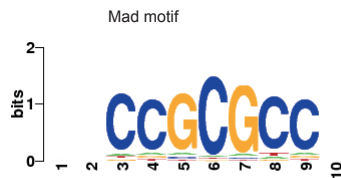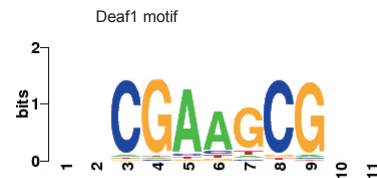

Supplement: Supplementary file 9 — Additional file 9. Motif analysis of EcR-enriched regions by MEME-ChIP and RSAT. a Motifs were enriched from EcR-binding sites of the arginine methyltransferase gene. b Average expression of the nearest genes of EcR. “EcR + EcRE” indicates EcR-peak regions enriched for the EcRE motif. “EcR − EcRE” indicated EcR-peak regions not enriched for EcRE motif. c Motifs enriched in distal EcR enhancer elements. [file 13072_2018_216_MOESM9_ESM.pdf]
